# Different rehabilitation destinations: non-clinical factors associated with inpatient rehabilitation versus skilled nursing placement

**DOI:** 10.3389/fresc.2026.1984193

**Published:** 2026-10-14

**Authors:** Heather A. Hayes, Chong Zhang, Siqi Xiang, Bradley Smith, Perry Williams, Angela P. Presson, Maggie French

**Affiliations:** 1 Department of Physical Therapy (AZ), Southern California University of Health Sciences, Whittier, CA, United States; 2 Study Design and Biostatistics Center, University of Utah, Salt Lake City, UT, United States; 3 Department of Occupational Therapy and Recreational Therapies, University of Utah, Salt Lake City, UT, United States; 4 Department of Physical Therapy and Athletic Training, University of Utah, Salt Lake City, UT, United States; 5 Department of Physical Therapy, Intermountain Health, Park City, UT, United States

**Keywords:** discharge planning, inpatient rehabilitation facility, post-acute care, rehabilitation, skilled nursing facility, stroke

## Abstract

**Purpose:**

Examine clinical and non-clinical factors associated with post-acute rehabilitation placement after acute ischemic stroke.

**Methods:**

236 adults hospitalized with acute ischemic stroke; independent and community-dwelling before stroke; and discharged to an inpatient rehabilitation facility (IRF; *n* = 171) or skilled nursing facility (SNF; *n* = 65). Clinical factors included NIHSS, Charlson Comorbidity Index (CCI), length of stay (LOS), AM-PAC “6-Clicks,” and neglect. Non-clinical factors included demographics and contextual characteristics. Logistic regression was used for associations with discharge location.

**Results:**

Individuals discharged to IRFs were younger, more likely to cohabitate, and had shorter LOS. Functional status (AM-PAC) and CCI did not differ significantly between groups despite differences in discharge destination. In univariable analyses, younger age, cohabitating marital status, living with family, available assistance, shorter LOS, private insurance, and higher NIHSS were associated with greater odds of IRF discharge. In the multivariable analysis, increasing age (OR 0.95, 95% CI 0.91–0.98) and greater comorbidity burden (CCI: OR 0.85, 95% CI 0.73–0.98) were associated with lower odds of IRF discharge. Marital status was not associated with discharge destination after adjustment.

**Conclusions:**

Among previously independent stroke survivors, discharged to an IRF or SNF increasing age and greater comorbidity burden were associated with lower odds of IRF placement after adjustment. NIHSS and AM-PAC Daily Activities scores were not associated with placement in the adjusted analysis.

## Introduction

Stroke is a leading cause of long-term disability in the United States, and the number of survivors requiring post-acute rehabilitation care (PARC) is expected to increase in the coming decades (1). The early recovery period, particularly the ﬁrst 90 days post-stroke, is critical, as access to intensive rehabilitation during this window can optimize functional recovery and reduce long-term disability (2). Discharge planning from acute hospitalization, speciﬁcally whether individuals post-stroke transition to an inpatient rehabilitation facility (IRF) or skilled nursing facility (SNF), is a pivotal decision with profound functional and economic consequences, as these settings differ substantially in rehabilitation intensity during the critical early post-stroke recovery period (3).

Clinical guidelines recommend IRF care over SNF care for individuals post-stroke, as IRF treatment is consistently associated with greater functional improvement, higher rates of community discharge, lower readmission, and lower mortality (4–6) Despite these recommendations, a larger proportion of stroke survivors in the United States are discharged to SNFs rather than IRFs (4). This is at least in part due to policy guidelines for determining discharge to an IRF versus SNF. Speciﬁcally, IRFs require that individuals are able to tolerate intensive multidisciplinary therapy (≥3 h/day, 5–6 days/week) and have the capacity for measurable functional improvement and community reintegration, whereas SNFs provide less intensive therapy coupled with nursing care (4). However, implementation of these recommendations varies considerably across institutions and regions, resulting in discharge practices that are inﬂuenced by non-clinical, hospital-level, and sociodemographic factors in addition to patient impairment severity (3, 6–8).

Although policy requirements partially shape discharge placement, substantial variability in referral patterns persists even among individuals with similar stroke severity and functional impairment. However, prior studies have often examined individual clinical (9) (e.g., stroke severity, comorbidities, and functional status) or sociodemographic (10–13) (age, sex, race) predictors of post-acute placement without determining whether contextual factors remain associated with IRF versus SNF placement among patients with comparable pre-stroke independence after accounting for standardized measures of both stroke severity and functional status.

Although prior studies have identified clinical, demographic, and contextual factors associated with post-acute rehabilitation placement after stroke, less is known about whether non-clinical factors remain associated with IRF versus SNF placement among patients with comparable pre-stroke independence when objective measures of stroke severity and functional status are considered concurrently. This distinction is important because differences in pre-stroke function or measured rehabilitation need may otherwise explain apparent differences in post-acute placement. By restricting the cohort to individuals who were independent and community dwelling before stroke and incorporating standardized measures of stroke severity, comorbidity, and function alongside demographic and contextual characteristics, this study examines whether variation in rehabilitation placement persists when important clinical indicators of rehabilitation need are accounted for.

Therefore, the aim of this study was to examine clinical and non-clinical (demographics and contextual) factors associated with discharge location to either an IRF or SNF among individuals hospitalized with acute ischemic stroke who were independent and community-dwelling prior to stroke onset. We hypothesized that non-clinical factors would demonstrate independent associations with discharge destination, even after accounting for clinical indicators of stroke severity.

## Methods

### Study design and cohort selection

This study was performed at the University of Utah, Salt Lake City, Utah. The IRF cohort was collected prospectively and included individuals admitted to the acute care hospital with a confirmed diagnosis of ischemic stroke verified by imaging (CT or MRI). The IRF participants provided informed consent and authorization under IRB_00114762. Data were collected through direct participant assessment and medical record review.

Data for the SNF cohort were collected retrospectively from the University of Utah Enterprise Data Warehouse under IRB_00171867, with a waiver of informed consent. The SNF cohort was collected retrospectively because prospective recruitment was disrupted during 2020–2022 by the COVID-19 pandemic. The same clinical, demographic, and contextual variables used for the IRF cohort were obtained from the available clinical record for the SNF cohort spanning the same timeframe January 1, 2020 to July 31, 2024. The SNF sample included individuals admitted into the acute care hospital under Neurology or Internal Medicine with a diagnosis of ischemic stroke (ICD-9-CM codes 433-434, 436; ICD-10 code I63.9) listed in the top three provider billing diagnoses.

For both cohorts, we included adult individuals (≥18 years) hospitalized with an acute ischemic stroke who were discharged from acute care to either an IRF or SNF. We included only individuals with a pre-stroke level of function (PLOF) characterized as independent and who were living in the community prior to their stroke (i.e., not residing in long-term care or other institutional settings), which was taken from chart review as part of routine questioning by the physical and/or occupational therapist and recorded in a discrete ﬁeld.

For both cohorts, we excluded individuals that were incarcerated, and those missing an NIHSS assessment. The ﬁnal analytic sample included 236 individuals, 171 discharged to an IRF and 65 to a SNF (Supplementary Figure S1). Data collection spanned

from January 1, 2020 to July 31, 2024 for both the IRF (prospective data) and SNF (retrospective data) cohorts.

### Variables and measures

The primary outcome was discharge location from the acute hospital (IRF vs. SNF). Clinical factors were deﬁned as variables reﬂecting stroke severity, comorbidity burden, and functional status during acute hospitalization. The clinical factors represent the patient's clinical presentation at the time of admission and includes 7 variables; 1) National Institutes of Health Stroke Scale (NIHSS); 2) Charlson Comorbidity Index (CCI); 3) acute care clinical length of stay (LOS); (4–6) functional presentation based on the three domains of the Activity Measure for Post-Acute Care (AM-PAC) scores: Basic Mobility (BM), Daily Activities (DA), and Applied Cognition (AC); and (7) presence of neglect or not [present, absent or missing]. The NIHSS is a rating of stroke severity, evaluating 11 areas and scores can range from 0 (no stroke) to 42 (severe stroke symptoms). Neglect was identiﬁed from the NIHSS (item 10) and clinical chart reviews. The AM-PAC “6-Clicks” was taken from chart review and is required to be performed by Physical and Occupational Therapy services. The raw score ranges from 6 to 24, with higher scores indicating greater independence.

Non-clinical factors were deﬁned as demographic and contextual characteristics not directly reﬂective of neurologic impairment or functional status. The non-clinical factors that we examined included 3 demographic variables and 5 contextual variables.

Demographic variables included: 1) age [years]; 2) sex [female or male]; 3) race [white or other/unknown]. Demographic variables were obtained from discrete ﬁelds in the chart for both cohorts. The contextual variables included: 1) marital status; 2) primary insurance; 3) home layout; 4) available assistance; 5) who patient lives with. Marital status was coded as alone [if divorced, separated, single, or widowed] or cohabitates [life partner or married] or missing. Primary insurance type was coded as Private, Medicare, Medicare Advantage (MA), Medicaid, or none. Home layout data was deﬁned as structural barriers present [stairs in the living setting, stairs to get into home, narrow doors, and/or limited space for equipment] or not present or missing. Available assistance was coded as yes when the clinical record documented that full-time or part-time physical assistance or supervision was available to the patient after discharge; otherwise, it was coded as no or missing. Living arrangement was coded as living alone or living with family/other, with the latter category including living with family or a paid helper. Unknown living arrangements were coded as missing. Contextual variables were obtained from discrete ﬁelds in the chart for both cohorts.

### Statistical analysis

Descriptive statistics were calculated for all variables stratiﬁed by discharge location. Continuous variables were compared using the Wilcoxon rank-sum test, and categorical variables using Chi-squared or Fisher's exact tests, as appropriate.

Univariable logistic regression was used to assess the unadjusted association between each factor and discharge location. Variables with *p* < 0.10 in the univariable analyses were considered for inclusion in the multivariable logistic regression model. Acute-care length of stay was excluded from the multivariable model because it may be influenced by processes occurring during discharge planning, including referral timing, insurance authorization, facility acceptance, and bed availability, and therefore may not clearly precede the final discharge destination. Collinearity among variables was evaluated using variance inﬂation factors (VIFs) with GVIF^1/2df^ > 2 considered suggestive of multicollinearity. Because the number of variables was limited by the number of individuals, only one variable was selected from related and correlated variables, prioritizing conceptual relevance and completeness of data. Missing data were handled by listwise deletion. Missingness was summarized for each model variable and by discharge destination. Participants included in the complete-case multivariable analysis were compared with those excluded because of missing model data. As a sensitivity analysis, missing Home Layout values were retained as an “unknown” category, and the multivariable logistic regression model was refitted using the expanded analytic sample. Model results were reported as odds ratios (OR) with 95% conﬁdence intervals (CI) and *p*-values. All analyses were conducted in R v4.3.127 (R Core Team, 2023) using two-tailed tests. Statistical signiﬁcance was evaluated at the 0.05 level.

## Results

Of the 236 individuals included in the analysis, 171 (72%) were discharged to an IRF and 65 (28%) to a SNF. Compared with individuals discharged to a SNF, those discharged to an IRF were younger (mean age 64 vs 74 years, *p* < 0.001), more likely to cohabitate (*p* = 0.006), had shorter LOS (mean 7.8 vs 13 days, *p* < 0.001), and had higher NIHSS scores (mean 10.0 vs 7.7 points, *p* = 0.006). Additional group differences were observed in insurance type, barriers in home layout, living status (Table 1).

**Table 1:** Descriptive summary of individuals post-stroke.

Variable	SNF (*N* = 65)	IRF (*N* = 171)	*P*-value
Non-Clinical Characteristics, Demographic			
Age at visit - Mean (SD)	74 (11)	64 (14)	<0.001^a^
Median (IQR)	75 (65, 82)	66 (55, 74)	-
Sex - Female	34 (52%)	75 (44%)	0.24^b^
Male	31 (48%)	96 (56%)	-
Race - white	51 (78%)	151 (88%)	0.06^c^
other/unknown	14 (22%)	20 (12%)	-
Non-Clinical Characteristics, Contextual			
Marital status - alone	39 (63%)	73 (43%)	0.006^b^
cohabitates	23 (37%)	98 (57%)	-
Missing *n* (%)	3 (4.6%)	-	-
Primary insurance - Private	13 (20%)	70 (41%)	0.005^c^
Medicare	24 (37%)	51 (30%)	-
Medicare Advantage (MA)	22 (34%)	28 (16%)	-
Medicaid	6 (9.2%)	20 (12%)	-
None	0 (0%)	2 (1.2%)	-
Home layout – barriers present	44 (69%)	136 (84%)	0.011^b^
Not present	20 (31%)	26 (16%)	-
Missing *n* (%)	1 (1.5%)	9 (5.3%)	-
Available assistance (yes) (no)	41 (79%) 11 (21%)	145 (91%) 14 (8.8%)	0.017^b^
Missing *n* (%)	13 (20%)	12 (7%)	-
Lives with - Lives alone	27 (42%)	41 (24%)	0.007^b^
Lives with family/other	37 (58%)	129 (76%)	-
Missing *n* (%)	1 (1.5%)	1 (0.58%)	-
Clinical Characteristics			
LOS - Mean (SD)	13 (11)	7.8 (5.7)	<0.001^a^
Median (IQR)	8.8 (6.0, 14)	6.0 (4.0, 9.5)	-
CCI score - Mean (SD)	4.6 (2.8)	3.9 (1.8)	0.14^a^
Median (IQR)	5.0 (2.0, 7.0)	4.0 (3.0, 5.0)	-
Missing *n* (%)	-	12 (7%)	-
NIHSS - Mean (SD)	7.7 (6.6)	10 (7.0)	0.006^a^
Median (IQR)	6.0 (2.0, 13)	8.0 (5.0, 15)	-
AM-PAC BM - Mean (SD)	13 (4.9)	12 (4.2)	0.18^a^
Median (IQR)	12 (8.0, 18)	12 (8.0, 16)	-
AM-PAC DA - Mean (SD)	13 (5.0)	12 (4.1)	0.11^a^
Median (IQR)	13 (9.0, 18)	12 (9.0, 15)	-
AM-PAC AC - Mean (SD)	14 (6.0)	14 (6.4)	0.73^a^
Median (IQR)	14 (8.0, 19)	13 (8.0, 20)	-
Neglect – absent Present missing	42 (74%) 15 (26%) 8 (12%)	111 (65%) 60 (35%) -	0.22^b^--

a Wilcoxon rank sum test, ^b^Chi-squared test, ^c^Fisher's exact test.

For categorical variables with missing data, percentages for observed categories were calculated using the number of participants with available data for that variable; missing percentages were calculated using the total number of participants in each discharge group (SNF, *N* = 65; IRF, *N* = 171). Statistical comparisons were based on nonmissing observations.

In univariable logistic regression (Table 2), several non-clinical (demographic and contextual) factors were associated with increased odds of discharge to an IRF, including cohabitating marital status (OR 2.28, 95% CI 1.26–4.19), living with family (OR 2.30, 95% CI 1.25–4.22), available assistance (OR 2.78, 95% CI 1.15–6.58), and private insurance (OR 2.53 vs. Medicare, 95% CI 1.20–5.58). Higher NIHSS was also associated with increased odds of IRF discharge (OR 1.06, 95% CI 1.01–1.11). In contrast, increasing age (OR 0.94 per year, 95% CI 0.92–0.97) and longer clinical LOS (OR 0.92 per day, 95% CI 0.88–0.96) were associated with reduced odds of discharge to an IRF. Since three contextual variables; 1) marital status, 2) available assistance, and 3) who individuals lived with were related, we chose marital status as the representative of these three variables for our multivariable model. In the multivariable logistic regression model excluding acute-care length of stay (*N* = 211; SNF=61, IRF=150), increasing age was associated with lower odds of IRF discharge (OR 0.95 per year, 95% CI 0.91–0.98; *p* = 0.002). Greater comorbidity burden was also associated with lower odds of IRF discharge (CCI: OR 0.85 per point, 95% CI 0.73–0.98; *p* = 0.034). Marital status was not statistically significant after adjustment (OR 1.79, 95% CI 0.88–3.66; *p* = 0.11). NIHSS, AM-PAC Daily Activities, insurance type, and home layout were also not associated with discharge destination in the adjusted model.

**Table 2:** Univariable and multivariable logistic regression models predicting discharge to an inpatient rehabilitation facility.

Variable	OR (univariable)	*P*-value	OR (multivariable)^b^	*P*-value
Non-Clinical Characteristics, Demographic				
Age	0.94 (0.92, 0.97)	<0.001	0.95 (0.91,0.98)	0.002
Sex (Male)	1.40 (0.79, 2.50)	0.25	-	-
Race (Other/unknown)	0.48 (0.23, 1.04)	0.06	-	-
Non-Clinical Characteristics, Contextual				
Marital Status (cohabitates vs. alone)	2.28 (1.26, 4.19)	0.007	1.79 (0.88, 3.66)	0.11
Primary Insurance^a^ (Private vs Medicare) (MA vs Medicare) (Medicaid vs Medicare)	2.53 (1.20, 5.58) 0.60 (0.28, 1.26) 1.57 (0.58, 4.74)	0.017 0.17 0.39	0.82 (0.31, 2.18) 0.49 (0.21, 1.15) 0.40 (0.09, 1.69)	0.69 0.11 0.20
Home Layout (not present)	0.42 (0.21, 0.83)	0.012	0.58 (0.26, 1.30)	0.18
Available Assistance (Yes)	2.78 (1.15, 6.58)	0.020	-	-
Lives with (family/other)	2.30 (1.25, 4.22)	0.007	-	-
Clinical Characteristics				
LOS	0.92 (0.88, 0.96)	<0.001		
CCI Score	0.87 (0.76, 0.99)	0.043	0.85 (0.73, 0.98)	0.034
NIHSS	1.06 (1.01, 1.11)	0.014	1.04 (0.99, 1.11)	0.16
AM-PAC BM	0.95 (0.89, 1.02)	0.14	-	-
AM-PAC DA	0.94 (0.88, 1.01)	0.09	0.99 (0.90, 1.08)	0.81
AM-PAC AC	1.01 (0.96, 1.06)	0.74	-	-
Neglect (present)	1.51 (0.79, 3.02)	0.22	-	-

a For two individuals with no insurance, we assigned NA to their insurance values so that they were excluded from the evaluation of insurance types.

b Sample size for the multivariable regression model: *N* = 211 (SNF = 61, IRF = 150).

The complete-case multivariable analysis included 211 participants; 25 were excluded because of missing information in one or more model variables, including CCI (*n* = 12), home layout (*n* = 10), marital status (*n* = 3), and insurance category (*n* = 2). Because some participants had missing information for more than one variable, these counts are not mutually exclusive. Of the excluded participants, 21 were discharged to an IRF and four to a SNF. No statistically significant differences were detected between included and excluded participants, although the small excluded group limited the precision of these comparisons (Supplementary Table S1).

In the sensitivity analysis, retaining missing Home Layout values as an “unknown” category increased the multivariable analytic sample from 211 to 219 participants. The principal findings were similar to the complete-case analysis: increasing age remained associated with lower odds of IRF discharge, as did greater comorbidity burden. Marital status and AM-PAC Daily Activities remained nonsignificant (Supplementary Table S2).

## Discussion

In this study of individuals hospitalized with acute ischemic stroke who were living independently in the community prior to hospitalization, we examined clinical and non-clinical (demographic and contextual) factors associated with discharge to either an IRF or a SNF. In this cohort of previously independent, community-dwelling individuals hospitalized with acute ischemic stroke, increasing age and greater comorbidity burden were associated with lower odds of IRF discharge in the adjusted analysis. Although marital status, available assistance, living arrangement, insurance type, stroke severity, and length of stay were associated with discharge destination in univariable analyses, these associations did not persist in the revised multivariable model. These findings suggest that age and comorbidity burden were the factors most consistently associated with the observed IRF-versus-SNF destination after accounting for the other variables represented in the model. These findings partially supported our hypothesis: age, a demographic characteristic, remained associated with discharge destination after adjustment, whereas the measured contextual characteristics did not.

First, younger individuals were signiﬁcantly more likely to be discharged to an IRF after adjustment for stroke severity and the other variables included in the model. Although age is consistently associated with poorer post-stroke outcomes (6, 7, 12, 14) these differences may partly reﬂect unequal access to high-intensity rehabilitation, given the documented beneﬁts of IRF care (3, 4, 15, 16). Older adults are more frequently discharged to SNFs, where recovery trajectories are typically less favorable (3). Thus, age may reﬂect not only biological variation in recovery potential, but also structural differences in post-acute care allocation. In practice, older age may be implicitly interpreted as reduced ability to tolerate intensive rehabilitation despite limited evidence that chronological age alone accurately reﬂects rehabilitation capacity or potential beneﬁt from high-intensity therapy. However, because we measured final discharge destination rather than initial clinical recommendations, insurance authorization, facility acceptance, bed availability, or patient and family preferences, the mechanisms underlying the association with age cannot be determined from the present data.

Second, cohabitating marital status, available assistance, and living with family or others were associated with greater odds of IRF discharge in the univariable analyses. However, marital status was not statistically significant after adjustment in the revised model excluding length of stay. These findings suggest that measures of social support may be related to placement in unadjusted analyses, but the present study does not establish an association independent of the other variables included in the model.

Third, greater comorbidity burden was associated with lower odds of IRF discharge after adjustment. Each one-point increase in CCI was associated with approximately 15% lower odds of IRF discharge. Although this finding may reflect differences in medical complexity or perceived ability to participate in intensive rehabilitation, the available data do not identify the specific clinical or placement processes underlying this association.

The complete-case analysis included 211 of 236 participants. Twenty-five participants were excluded because of missing information in one or more model variables. No statistically significant differences were detected between included and excluded participants for the measured demographic, clinical, functional, or contextual characteristics, although the small excluded group limited the precision of these comparisons (Supplementary Table S1). When missing Home Layout values were retained as an “unknown” category, the analytic sample increased to 219 participants. The direction, magnitude, and statistical interpretation of the principal estimates were similar to those of the complete-case model.

Notably, none of the AM-PAC domains differed significantly between the IRF and SNF groups in unadjusted comparisons. In the multivariable analysis, the AM-PAC Daily Activities domain was not independently associated with discharge location. Because Basic Mobility and Applied Cognition were not represented in the multivariable model, adjusted conclusions regarding functional status should be limited to the Daily Activities domain. Age was the only measured non-clinical characteristic that remained associated with IRF versus SNF discharge destination after adjustment. Although insurance demonstrated associations in unadjusted models, consistent with prior research (6), it did not remain signiﬁcant after adjustment, possibly reﬂecting limited statistical power.

Collectively, these findings indicate that increasing age and greater comorbidity burden were associated with lower odds of IRF discharge after adjustment. The contextual characteristics associated with placement in the univariable analyses did not remain statistically significant in the revised multivariable model. Because the study measured final discharge destination rather than the sequence of referral, authorization, acceptance, and patient or family decision-making, the results should be interpreted as associations with observed placement rather than evidence of causal effects on placement decisions. Although IRF admission criteria emphasize medical stability, tolerance for intensive interdisciplinary rehabilitation, and anticipated functional improvement (17) how these criteria are operationalized in clinical practice may vary across institutions and clinicians. Greater transparency and evidence-informed frameworks for discharge planning may help ensure equitable access to intensive rehabilitation services.

## Limitations

This study has several limitations. First, all data was captured from within a single health system, which may limit generalizability to other regions or practice environments. Second, racial differences in discharge destination could not be robustly evaluated because our sample was predominantly White, reﬂecting regional demographics. Prior research has identiﬁed racial disparities in post-acute placement, warranting further investigation in more diverse cohorts. Third, the two cohorts differed in their method of data collection: the IRF cohort was enrolled prospectively, whereas the SNF cohort was identified retrospectively from the Enterprise Data Warehouse because prospective recruitment was disrupted by the COVID-19 pandemic. Although the same study variables were obtained for both cohorts, retrospective data collection in the SNF cohort resulted in greater missingness for select variables and may have introduced information or selection bias. Fourth, the overall sample size constrained the number of predictors that could be included in multivariable modeling and limited statistical power to detect smaller effects. As such, ﬁndings should be interpreted with caution and validated in larger, multi-center samples. Fifth, the adjusted analysis excluded 25 participants with missing information in one or more model variables. Although included and excluded participants did not differ significantly on the measured characteristics and the primary estimates were stable when missing Home Layout was retained as an unknown category, the small excluded group limited these comparisons, and bias related to missing data cannot be ruled out.

Finally, although we included established measures of stroke severity, comorbidity burden, and functional status, we did not capture all clinical or logistical factors that may inﬂuence discharge decisions. Variables such as medical complexity requiring specialized equipment (e.g., ventilatory support, dialysis, complex wound care), bed availability, or clinician assessment of medical stability were not available and may explain discharge decisions not fully captured by the variables available in this analysis.

## Conclusions

Among previously independent stroke survivors discharged to an IRF or SNF, increasing age and greater comorbidity burden were associated with lower odds of IRF placement after adjustment. AM-PAC Daily Activities scores and stroke severity were not associated with placement in the adjusted analysis, and the observed univariable association with marital status did not persist after adjustment. Additional studies incorporating referral recommendations, insurance authorization, facility acceptance, bed availability, and patient or family preferences are needed to clarify the processes contributing to post-acute rehabilitation placement. These ﬁndings suggest that real-world post-acute rehabilitation placement may not consistently align with measurable indicators of rehabilitation need alone. More transparent and evidence-informed discharge frameworks may help better align rehabilitation intensity with recovery potential after stroke.

## Data Availability

The datasets analyzed for this study are not publicly available because of institutional and ethical restrictions protecting participant privacy. Reprints are available upon request. Requests to access the datasets should be directed to heatherhayes@scuhs.edu.
