# Supplementary material for: Different rehabilitation destinations: non-clinical factors associated with inpatient rehabilitation versus skilled nursing placement
Source: Front Rehabil Sci. 2026 Oct 14;7:1984193. doi: 10.3389/fresc.2026.1984193 (PMC13713781; doi:10.3389/fresc.2026.1984193)
Supplement: Supplementary file 1 [file Datasheet1.pdf]

Supplemental Table 1. Comparison of Participants Included in and Excluded From the Complete-Case Multivariable Analysis

Variable	Included (N = 211)	Excluded (N = 25)	P-value
**Discharge Destination -IRF**	150 (71%)	21 (84%)	0.17[c]
**-SNF**	61 (29%)	4 (16%)	
**Non-Clinical Characteristics, Demographic**			
**Age at visit -** Mean (SD)	66.83 (13.96)	65.49 (13.59)	0.65[e]
Median (IQR)	68.20 (57.98, 76.74)	66.34 (59.19, 76.58)	-
**Sex -** Female	102 (48%)	7 (28%)	0.054[c]
Male	109 (52%)	18 (72%)	-
**Race -** white	181 (86%)	21 (84%)	0.77[f]
other/unknown	30 (14%)	4 (16%)	-
**Non-Clinical Characteristics, Contextual**			
**Marital status -** alone	99 (47%)	13 (59%)	0.28[c]
cohabitates	112 (53%)	9 (41%)	-
Missing n (%)	0	3 (12%)	-
**Primary insurance -** Private	77 (36%)	6 (26%)	0.51[f]
Medicare	66 (31%)	9 (39%)	-
Medicare Advantage (MA)	46 (22%)	4 (17%)	-
Medicaid	22 (10%)	4 (17%)	-
None	0(0%)	2(8%)	-
**Home layout** – barriers present	166 (79%)	14 (93%)	0.32[f]
Not present	45 (21%)	1 (6.7%)	-
Missing n (%)	0	10 (40%)	-
**Available assistance** (yes)	168 (88%)	18 (90%)	1.00[f]
(no)	23 (12%)	2 (10%)	-
Missing n (%)	20 (9.5%)	5 (20%)	-
**Lives with -** Lives alone	61 (29%)	7 (29%)	0.99[c]
Lives with family/other	149 (71%)	17 (71%)	-
Missing n (%)	1 (0.47%)	1 (4%)	-
**Clinical Characteristics**			
**LOS -** Mean (SD)	9.4 (8.0)	7.3 (3.7)	0.44[e]
Median (IQR)	7.0 (4.9, 11)	6.0 (5.0, 9.0)	-
**CCI score -** Mean (SD)	4.2 (2.2)	3.2 (1.7)	0.22[e]
Median (IQR)	4.0 (3.0, 5.0)	4.0 (1.0, 5.0)	-
Missing n (%)	0	12 (48%)	-
**NIHSS -** Mean (SD)	9.5 (7.0)	9.2 (6.8)	0.88[e]
Median (IQR)	8.0 (3.5, 15)	7.0 (5.0, 12)	-
**AM-PAC BM -** Mean (SD)	12.13 (4.30)	13.64 (5.07)	0.14[e]
Median (IQR)	12 (8, 16)	15 (10, 17)	-
**AM-PAC DA -** Mean (SD)	13 (4.4)	12 (4.1)	0.56[e]
Median (IQR)	12 (9.0, 16)	12 (9.0, 14)	-
**AM-PAC AC -** Mean (SD)	14.24 (6.31)	13.48 (6.04)	0.58[e]
Median (IQR)	13 (8, 20)	13 (7, 18)	-

**Neglect** – absent	134 (66%)	19 (76%)	0.32[c]
Present	69 (34%)	6 (24%)	-
missing	8 (3.8%)	0	-

Note. Participants were excluded from the complete-case multivariable analysis when data were missing for one or more variables included in the final model. Percentages for observed categorical values were calculated using participants with available data; missing percentages were calculated using the total number of participants in each group. Continuous variables are reported on their original clinical scales.

Two participants with no insurance were treated as having unavailable insurance-category data for the multivariable model.

[c] chi-square test; [e] exact Wilcoxon rank-sum test; [f] Fisher's exact test.
