# Supplementary material for: Different rehabilitation destinations: non-clinical factors associated with inpatient rehabilitation versus skilled nursing placement
Source: Front Rehabil Sci. 2026 Oct 14;7:1984193. doi: 10.3389/fresc.2026.1984193 (PMC13713781; doi:10.3389/fresc.2026.1984193)
Supplement: Supplementary file 2 [file Datasheet2.pdf]

Supplemental Table 2. Sensitivity Analysis Treating Missing Home Layout Values as an Unknown Category.

Variable	OR (univariable)	P-value	OR (multivariable)**	P-value
**Non-Clinical Characteristics, Demographic**				
**Age**	0.94 (0.92, 0.97)	<0.001	0.95 (0.91, 0.98)	0.002
**Sex** (Male)	1.40 (0.79, 2.50)	0.25	-	-
**Race** (Other/unknown)	0.48 (0.23, 1.04)	0.06	-	-
**Non-Clinical Characteristics, Contextual**				
**Marital Status** (cohabitates vs. alone	2.28 (1.26, 4.19)	0.007	1.84 (0.92, 3.75)	0.09
**Primary Insurance***				
(Private vs Medicare)	2.53 (1.20, 5.58)	0.017	0.85 (0.32, 2.27)	0.75
(MA vs Medicare)	0.60 (0.28, 1.26)	0.17	0.51 (0.22, 1.20)	0.12
(Medicaid vs Medicare)	1.57 (0.58, 4.74)	0.39	0.43 (0.10, 1.83)	0.25
**Home Layout** (not present)	0.42 (0.21, 0.83)	0.012	0.58 (0.26, 1.30)	0.18
Home Layout *(Unknown)*	*2.91 (0.53, 54.44)*	*0.32*	*1.39 (0.19, 28.51)*	*0.78*
**Available Assistance** (Yes)	2.78 (1.15, 6.58)	0.020	-	-
**Lives with** (family/other)	2.30 (1.25, 4.22)	0.007	-	-
**Clinical Characteristics**				
**LOS**	0.92 (0.88, 0.96)	<0.001	-	-
**CCI Score**	0.87 (0.76, 0.99)	0.043	0.85 (0.73, 0.98)	0.03
**NIHSS**	1.06 (1.01, 1.11)	0.014	1.04 (0.98, 1.11)	0.17
**AM-PAC BM**	0.95 (0.89, 1.02)	0.14	-	-
**AM-PAC DA**	0.94 (0.88, 1.01)	0.09	0.99 (0.90, 1.08)	0.80
**AM-PAC AC**	1.01 (0.96, 1.06)	0.74	-	-
**Neglect** (present)	1.51 (0.79, 3.02)	0.22	-	-

**Sample size for the multivariable regression model: N=219

MA = Medicare Advantage, LOS = Acute length of stay, CCI =Charlson Comorbidity Index, NIHSS = National Institutes of Health Stroke Severity, AM-PAC = Activity Measure for Post-Acute Care, BM = Basic Mobility, DA = Daily Activity, AC = Applied Cognition
